# Postnatal PTSD: Risks and Consequences

**DOI:** 10.1192/j.eurpsy.2022.99

**Published:** 2022-09-01

**Authors:** W. El-Hage

**Affiliations:** Université de Tours, Centre Régional De Psychotraumatologie Cvl, Tours, France

**Keywords:** postnatal, Childbirth, Post-traumatic stress disorder

## Abstract

Post-traumatic stress disorder (PTSD) occurs in 4% of all pregnancies during the postnatal period. This prevalence can increase in high-risk groups reaching a mean prevalence of 18%. Some risk factors are significantly associated with the development or exacerbation of postnatal PTSD, including prenatal depression and anxiety, pre-pregnancy history of psychiatric disorders, history of sexual trauma, intimate partner violence, emergency childbirth, distressing events during childbirth and psychosocial attributes. Maternal postnatal PTSD is highly associated with the difficulties in mother-infant bond and the postpartum depression. Evidence shows significant links between psychological, traumatic and birth-related risk factors as well as the perceived social support and PTSD following childbirth. The City Birth Trauma Scale can be recommended as a universal instrument for diagnosis of postnatal PTSD.

**Disclosure:**

Wissam El-Hage reports personal fees from Air Liquide, EISAI, Janssen, Lundbeck, Otsuka, UCB and Chugai.

